# Higher Neighborhood Crime Rates Don't Always Predict Early Initiation of Tobacco, Marijuana, and Alcohol

**DOI:** 10.31586/jsmhes.2024.1049

**Published:** 2024-08-24

**Authors:** Shervin Assari, Payam Sheikhattari

**Affiliations:** 1 Charles R Drew University of Medicine and Science, Los Angeles, California, USA; 2 Morgan State University, Baltimore, Maryland, USA

**Keywords:** Adolescent Substance Use, Environmental Crime Statistics, Tobacco Use, Marijuana Use, Alcohol Use, Drug-Related Crime

## Abstract

**Background:**

The initiation of substance use during adolescence is a significant public health concern with long-term implications. Structural and environmental factors, such as community-level crime statistics related to drug offenses, are often assumed to influence the likelihood of substance use among youth. However, the relationship between these environmental crime indicators and early substance use initiation in adolescents is not well understood.

**Objective:**

This study aims to examine the association between environmental drug-related crime statistics—derived from Uniform Crime Reports, including drug abuse violations, drug sales, marijuana sales, drug possession, and driving under the influence (DUI)—and the use of tobacco, marijuana, and alcohol among adolescents aged 9-16 in the United States.

**Methods:**

Data from the Adolescent Brain Cognitive Development (ABCD) study, which follows a cohort of adolescents from ages 9-10 to 16, were analyzed. 11,878 participants entered our analysis. The primary environmental measures included total drug abuse violations, drug sale offenses, marijuana sale offenses, drug possession offenses, and DUI reports. Substance use outcomes of interest were the frequency and prevalence of tobacco, marijuana, and alcohol use across the observed age range.

**Results:**

Contrary to expectations, higher levels of drug-related crime in a community were not associated with increased use of tobacco, marijuana, or alcohol among adolescents. In fact, the study found a significant inverse relationship between the total number of drug-related crimes and tobacco use, suggesting lower tobacco use in areas with higher reported drug abuse violations, drug sales, marijuana sales, drug possession, and DUI incidents. No significant associations were observed between these crime indicators and the use of marijuana or alcohol.

**Conclusions:**

These findings challenge the prevailing assumption that higher environmental drug-related crime statistics necessarily predict greater substance use among adolescents. The observed inverse relationship between drug-related crime and tobacco use warrants further investigation to understand the underlying mechanisms and to inform targeted intervention strategies. Future research should explore the complex interplay between structural environmental factors and youth substance use to better inform public health policies.

## 1. Introduction

Substance use during adolescence poses a significant public health challenge, with enduring consequences for both individual health and broader societal well-being [1,2]. Understanding the factors that contribute to the early initiation and sustained use of substances such as tobacco, marijuana, and alcohol is crucial for developing effective prevention strategies [3,4]. Among the numerous influences on adolescent substance use, structural and environmental factors have garnered increasing attention [5]. These factors, which encompass community-level characteristics like crime rates, socioeconomic conditions, and the density of tobacco and liquor retail outlets, are believed to shape the environments in which young people are exposed to and initiate substance use [6].

The impact of structural and environmental influences on various health behaviors, including substance use, is well-documented [7-10]. For instance, higher crime rates, particularly those related to drug activities, are often presumed to create environments that elevate the likelihood of substance use among adolescents [11]. The underlying assumption is that areas with elevated drug-related crime may expose youth to greater opportunities for substance use, normalize drug-related behaviors, or reflect broader social and economic distress that predisposes young people to engage in substance use as a coping mechanism [12-14].

However, while the association between structural and environmental influences on substance use is well-established in adult populations, the extent to which these higher-level factors affect the early initiation of substance use among youth remains less clear [12-14]. Adolescents are at a critical and formative stage of development, and the pathways through which environmental risk factors influence their behaviors may differ substantially from those observed in adults^2^. For example, research has shown that for adults, drug-related offenses—such as drug abuse violations, drug sales, marijuana sales, drug possession, and driving under the influence (DUI)—are associated with higher rates of substance use [15-17]. However, the specific role of these drug-related crime statistics in predicting substance use among adolescents is not fully understood.

## 2. Aims

This study utilizes data from the Adolescent Brain Cognitive Development (ABCD) study [18-22] to examine the influence of environmental drug-related crime statistics on the subsequent use of tobacco, marijuana, and alcohol among adolescents aged 9-10 to 16 in the United States. We hypothesized that adolescents residing in areas with higher levels of drug offenses—including drug sales, marijuana sales, drug possession, and DUI incidents—would exhibit higher rates of tobacco, marijuana, and alcohol use. This paper aims to explore these associations in detail, considering the broader implications for understanding the environmental and structural determinants of adolescent substance use and the potential for targeted policy interventions in areas with elevated substance use or crime rates.

## 3. Methods

### 3.1. Design and Setting

This study employed a longitudinal design using data from the Adolescent Brain Cognitive Development (ABCD) study [18-22], the largest long-term investigation of brain development and child health in the United States. The ABCD study follows a diverse cohort of children from ages 9-10 through early, mid, and late adolescence, with the goal of understanding various factors influencing brain development and related outcomes, particularly the early initiation of substance use behaviors such as tobacco, marijuana, and alcohol. The data for this analysis were collected every six months over several years, enabling early detection of substance use initiation. This frequent measurement schedule also allowed for the examination of predictors and changes in substance use patterns as participants aged from 9-10 to 16 years old.

### 3.2. Sample and Sampling

The ABCD study recruited a nationally representative sample of 11,878 children aged 9-10 years from 21 research sites across the United States. The sample was drawn using a probability sampling method designed to reflect the demographic diversity of the U.S. population. This approach ensured the inclusion of participants from various racial, ethnic, socioeconomic, and geographical backgrounds, providing a broad and generalizable understanding of adolescent development. For the present analysis, we included participants who had data on substance use behaviors and environmental crime statistics from the baseline assessment through the 16-year follow-up.

### 3.3. Process and Measures

Substance use behaviors (tobacco, marijuana, and alcohol) were assessed through self-report questionnaires administered to participants every six months. These assessments were conducted either during in-person visits at research sites or via remote surveys, depending on participant availability and logistical considerations. The self-report format was chosen to capture the participants' perceptions and experiences of substance use in their own words, providing a subjective account of their behaviors. To ensure the accuracy and reliability of self-reported data, participants were assured of the confidentiality of their responses. Standardized questionnaires, such as the iSay Sipping Inventory [23] and the web-based Timeline Follow-Back [24], were used. These measures were administered using standardized protocols across all study sites. We used three dichotomous variables to reflect the use of tobacco, marijuana, and alcohol between the ages of 9 and 16, indicating early initiation.

In addition to substance use, environmental crime statistics were derived from Uniform Crime Reports [25-27], capturing indicators such as total drug abuse violations, drug sales, marijuana sales, drug possession, and DUI incidents in the participants' residential areas. These crime statistics were linked to participants' data based on their residential zip codes, providing a measure of the environmental risk factors present in their communities. We obtained these data from the ABCD residential history dataset. The variables used included 1) Total Drug Abuse Violation, 2) Drug Sale Offenses, 3) Marijuana Offenses, 4) Drug Possession Offenses, and 5) Driving Under the Influence (DUI) [28-31].

### 3.4. Data Analysis

Data were analyzed using Stata to assess the relationship between baseline environmental crime statistics and substance use behaviors over the follow-up period. Pearson correlations were employed for bivariate analyses and were specifically used in Tables 1, 2, and 3. Some of the statistics reported in these tables may overlap. A significance level of p < 0.05 was set for all analyses.

### 3.5. Ethics

The ABCD study received ethical approval from the Institutional Review Boards (IRBs) of the University of California, San Diego (UCSD), as well as from the IRBs of the 20 other participating research sites. Informed consent was obtained from the parents or legal guardians of all youth participants, and assent was obtained from the youth participants themselves. The ethical considerations of this study were aligned with the principles outlined in the Declaration of Helsinki and the U.S. Department of Health and Human Services regulations for the protection of human research subjects. Data were kept fully confidential, and all participants were informed of their right to withdraw from the study at any time without penalty. The confidentiality and anonymity of all collected data were strictly maintained, with identifying information removed and data securely stored in accordance with federal regulations and IRB guidelines.

## 4. Results

### 4.1. Correlations between Crime Data

As shown in Table 1, there were strong positive correlations between the different types of crime data reported in the ABCD study. Specifically, total drug abuse violations were highly correlated with drug sale offenses (r = 0.987; p < 0.001), marijuana offenses (r = 0.965; p < 0.001), drug possession offenses (r = 0.999; p < 0.001), and DUI incidents (r = 0.977; p < 0.001). Similarly, drug sale offenses were strongly correlated with marijuana offenses (r = 0.976; p < 0.001), drug possession offenses (r = 0.980; p < 0.001), and DUI incidents (r = 0.967; p < 0.001). All other crime categories showed significant positive correlations with each other, indicating a high level of overlap among these crime statistics.

### 4.2. Correlations between Substance Use Initiation in Youth

Table 2 presents the correlations between the initiation of different substances among youth. Early tobacco initiation showed a significant positive correlation with early initiation of both marijuana (r = 0.422, p < 0.001) and alcohol (r = 0.183, p < 0.001). Similarly, early marijuana initiation was positively correlated with early alcohol initiation (r = 0.141, p < 0.001). These findings suggest a clustering of early substance use, indicating that the initiation of one substance is associated with the initiation of others. Notably, the strongest association was observed between early tobacco and marijuana initiation.

### 4.3. Correlations between Crime Data and Substance Use Initiation in Youth

As indicated in Table 3, the correlations between crime data and substance use initiation were generally weak. Total Drug Abuse Violations were negatively correlated with Tobacco initiation (r = −0.029; p = 0.002) and Alcohol initiation (r = −0.011; p = 0.249), but showed no significant correlation with Marijuana initiation (r = 0.007; p = 0.443). Similarly, Drug Sale Offenses were negatively correlated with Tobacco initiation (r = −0.027; p = 0.005) and Alcohol initiation (r = −0.012; p = 0.206), with no significant correlation with Marijuana initiation (r = 0.007; p = 0.480). Marijuana Offenses were also negatively correlated with Tobacco initiation (r = −0.023; p = 0.015) and had no significant correlation with Marijuana (r = 0.007; p = 0.455) or Alcohol initiation (r = 0.003; p = 0.747). Drug Possession Offenses and DUI incidents followed similar patterns, showing negative correlations with Tobacco initiation and no significant correlations with Marijuana or Alcohol initiation.

## 5. Discussion

In this study, we aimed to explore the influence of baseline environmental drug-related crime statistics on the early initiation of tobacco, marijuana, and alcohol use among adolescents aged 9-16 in the United States. Specifically, we sought to determine whether indicators derived from Uniform Crime Reports—such as drug abuse violations, drug sales, marijuana sales, drug possession, and DUI at baseline—were predictive of higher subsequent substance use within this population. The results are intended to contribute to our understanding of how structural and environmental factors, particularly those related to crime, influence the early initiation and continued use of substances in youth.

Contrary to our expectations, the results did not indicate a positive association between higher baseline drug-related crime rates and increased initiation of any substances among US adolescents. No significant correlations were observed between the crime statistics and the use of marijuana or alcohol. Surprisingly, we found lower rates of tobacco use in areas reporting higher total drug offenses, drug sales, marijuana sales, drug possession, and DUI incidents. This inverse, weak, yet statistically significant relationship between these environmental crime indicators and tobacco use among ABCD adolescents was unexpected.

The literature on substance use has long suggested that ecological and structural factors, such as community crime rates, socioeconomic status, and neighborhood disorganization, play a significant role in shaping substance use behaviors [7,32]. Environments with higher crime rates, particularly drug-related offenses, are typically associated with increased exposure to substance use opportunities, normalization of risky behaviors, and overall higher substance use among adolescents. These findings have led to the widely held belief that individuals in high-crime areas are more likely to engage in substance use due to environmental stressors and increased accessibility to substances.

However, our study’s findings did not align with this prevailing understanding. Rather than observing higher youth substance use in areas with elevated drug-related crime^33,34^, we found no significant association for marijuana or alcohol use and an unexpected inverse relationship with tobacco use. This discrepancy raises questions about the applicability of existing theories to the specific age group and demographic context examined in the ABCD study. It suggests that the relationship between environmental crime factors and adolescent substance use may be more complex and potentially mediated by other variables not captured in our study [35-37].

Several potential explanations could account for the divergence between our findings and the existing literature. One possibility is that adolescents in high-crime areas may be subject to stricter parental or community supervision, reducing opportunities for substance use [32,38]. Additionally, these areas might have a more robust law enforcement presence or community programs aimed at substance use prevention, which could contribute to lower use rates. Moreover, as previous research by Luthar [39-48] has shown, substance use is increasingly prevalent in affluent areas, indicating that environmental influences may differ based on socioeconomic status. Another possibility is that our measures of drug-related crime did not adequately capture the nuances of the environments these adolescents live in, such as informal social controls or protective community factors that mitigate the risk of substance use. Finally, resilience factors, such as individual-level psychological or social support, might buffer the impact of adverse environments on substance use behaviors.

### 5.1. Future Research

Future research should aim to explore more deeply the mechanisms behind the lack of association between environmental crime data and adolescent substance use initiation. Studies could benefit from incorporating a broader range of ecological and individual-level variables, such as neighborhood cohesion, family dynamics, and access to substance use prevention resources, to better understand the complex interplay of factors. Longitudinal research designs that follow adolescents over an extended period may also provide more insight into how the influence of environmental crime evolves as youth age. Additionally, qualitative studies could investigate the lived experiences of adolescents in high-crime areas to uncover contextual factors that might not be captured through quantitative measures.

### 5.2. Limitations

This study has several limitations that should be acknowledged. First, relying solely on baseline Uniform Crime Reports does not fully capture the complexities of the environments in which adolescents live, as these reports are limited to recorded offenses and do not account for unreported crimes or the subjective experiences of residents. Additionally, the same environmental conditions may have different effects across groups, with environmental impacts potentially being buffered or modified by other factors at the family or individual level. Moreover, the short follow-up period of the study limits our ability to generalize our conclusions about the lack of a relationship between environmental crime and substance use. The generalizability of our findings may also be constrained by the non-random sample design of the ABCD study. While the ABCD demographic data reflect the U.S. population, the study included larger samples from certain geographic areas, such as California, which may limit the representativeness of our results for all U.S. adolescents. In addition, we only investigated overall associations. The effects of social context may differ for males, females, low-income, and high-income groups. Finally, this study only investigated the effect of crime data, without considering other contextual factors such as retail density, the availability of substances in neighborhoods, schools, and families, or substance use by parents. As a result, not all relevant contextual factors were exhaustively studied here.

## 6. Conclusions

In conclusion, our study challenges the assumption that higher environmental drug-related crime statistics predict increased substance use among adolescents. The unexpected finding of a weak yet statistically significant association between higher crime rates and lower tobacco use may be influenced by the large sample size. These results suggest that the relationship between structural factors and adolescent behavior is complex and warrants further investigation. Our findings highlight the need for a nuanced understanding of ecological influences on youth substance use and underscore the importance of considering a wide range of factors, including potential protective elements, in future research and intervention efforts.

## Figures and Tables

**Table 1.** Correlations between Crime Data in the ABCD Study

		1	2	3	4	5
**1 Total Drug Abuse Violation**	r	1				
	P					
**2 Drug Sale Offenses**	r	0.987	1			
	P	<0.001				
3 Marijuana Offenses	r	0.965	0.9755	1		
	P	<0.001	<0.001			
4 Drug Possession Offenses	r	0.9991	0.9803	0.9589	1	
	P	<0.001	<0.001	<0.001		
**5 Driving Under the Influence (DUI)**	r	0.977	0.9674	0.9759	0.9759	1
	P	<0.001	<0.001	<0.001	<0.001	

***Note:*** Data Came from the Adolescent Brain Cognitive Development (ABCD) study. Pearson correlations were used. N = 11,878.

**Table 2.** Correlations between Substance Use Initiation of Youth

		1	2	3
**1 Tobacco Initiation**	r	1		
	P			
**2 Marijuana Initiation**	r	0.422	1	
	P	<0.001		
**3 Alcohol Initiation**	r	0.1826	0.1414	1
	P	<0.001	<0.001	

***Note:*** Data Came from the Adolescent Brain Cognitive Development (ABCD) study. Pearson correlations were used. N = 11,878.

**Table 3.** Correlations between Crime Data and Substance Use Initiation of Youth

		1	2	3	4	5	6	7	8
**1 Tobacco Initiation**	r	1.000							
	P								
**2 Marijuana Initiation**	r	0.422	1.000						
	P	< 0.001							
**3 Alcohol Initiation**	r	0.183	0.141	1.000					
	P	< 0.001	< 0.001						
**4 Total Drug Abuse Violation**	r	−0.029	0.007	−0.011	1.000				
	P	0.002	0.443	0.249					
**5 Drug Sale Offenses**	r	−0.027	0.007	−0.012	0.987	1.000			
	P	0.005	0.480	0.206	< 0.001				
**6 Marijuana Offenses**	r	−0.023	0.007	0.003	0.965	0.976	1.000		
	P	0.015	0.455	0.747	< 0.001	< 0.001			
**7 Drug Possession Offenses**	r	0.029	0.007	−0.010	0.999	0.980	0.959	1.000	
	P	0.002	0.437	0.277	< 0.001	< 0.001	< 0.001		
**8 Driving Under the Influence (DUI)**	r	−0.027	0.008	−0.004	0.977	0.967	0.976	0.976	1.000
	P	0.004	0.405	0.702	0.000	< 0.001	< 0.001	< 0.001	

***Note:*** Data Came from the Adolescent Brain Cognitive Development (ABCD) study. Pearson correlations were used. N = 11,878.
